# 2-Ethyl-4-methyl-1*H*-imidazol-3-ium bromide

**DOI:** 10.1107/S2414314622011725

**Published:** 2022-12-15

**Authors:** Ray J. Butcher, Andrew P. Purdy

**Affiliations:** a Department of Chemistry, Howard University, 525 College Street NW, Washington DC 20059, USA; b Chemistry Division, Code 6123, Naval Research Laboratory, 4555 Overlook Av, SW, Washington DC 20375-5342, USA; University of Aberdeen, United Kingdom

**Keywords:** crystal structure, imidazolium cations, hydrogen bonding

## Abstract

In the title mol­ecular salt, C_6_H_11_N_2_^+^·Br^−^, the components are linked by N—H⋯Br⋯H—N hydrogen bonds into *C*(8)chains of alternating cations and anions propagating in the *b*-axis direction; these chains are cross-linked in the *c*-axis direction by weak C—H⋯Br hydrogen bonds.

## Abstract 2

In the title salt, the bromide ions accept N—H⋯Br hydrogen bonds from the cations, generating *C*(8) chains propagating in the *b*-axis direction.

## Graphical abstract

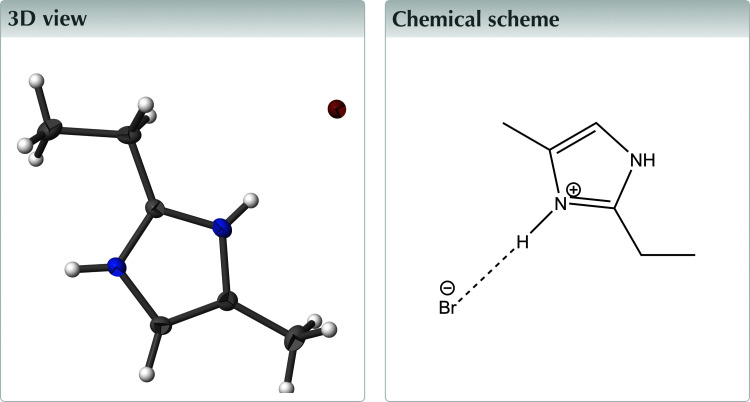

## Structure description

The unique structure of imidazole, containing two N atoms in a five-membered ring, permits it to accept a proton on one of its N atoms to form a cation and simultaneously deliver another proton from the other N atom to a suitable acceptor. In fact, this sort of shuttling action has been proposed as part of the catalytic mechanism of a number of enzymes (Mikulski & Silverman, 2010 ▸), and is consistent with the proton-conductivity properties of imidazole in the solid state where long hydrogen-bonded chains are present (Kawada *et al.*, 1970 ▸). These moieties and their derivatives have been implicated in proton-coupled electron-transfer processes (Huynh & Meyer, 2007 ▸; Onidas *et al.*, 2010 ▸). Consequently, there have been many theoretical (Scheiner & Yi, 1996 ▸; Kumar & Venkatnathan, 2015 ▸) and structural studies (Purdy *et al.*, 2007 ▸; Kim *et al.*, 2016 ▸) investigating these species. In this paper, we report a crystal structure containing the 2-ethyl-4-methyl-1*H*-imidazol-3-ium (C_6_H_11_N_2_^+^) cation. There have been four previous reports of structures containing this species (CSD refcode LEZSAL, Amanokura *et al.*, 2007 ▸; POJFOL, Beckett *et al.*, 2014 ▸; HOJJAT, Arici *et al.*, 2014 ▸; UMALAX, Kazimierczuk *et al.*, 2016 ▸).

**Figure 1:**
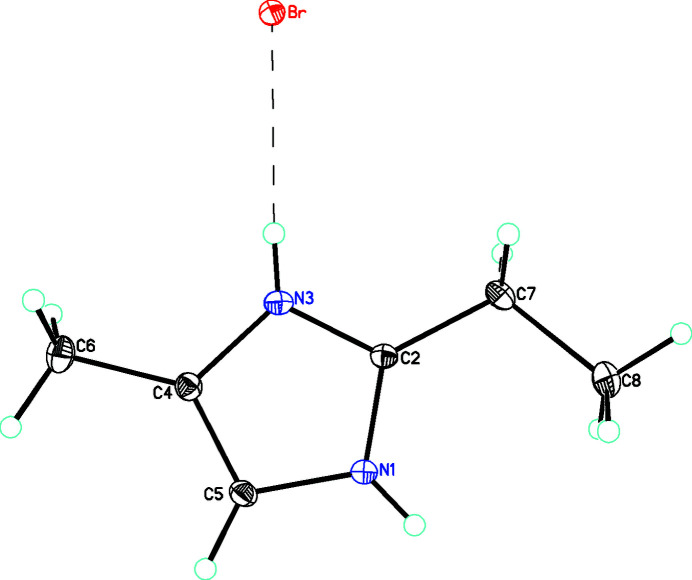
The mol­ecular structure of **1** showing 30% displacement ellipsoids. The hydrogen bond is shown with a dashed line.

**Figure 2:**
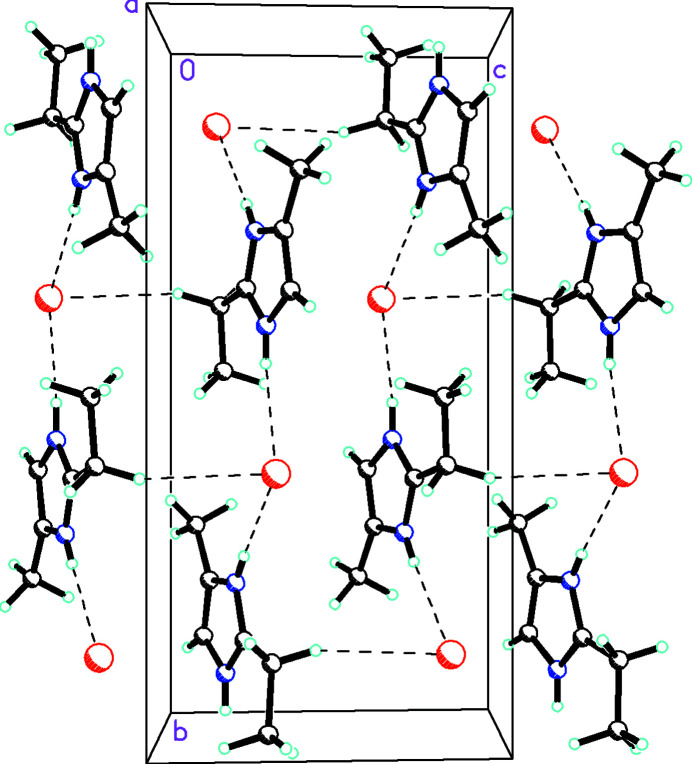
Packing diagram of **1** viewed down [100] showing how the cations and anions are linked into *C*(8)chains propagating in the *b*-axis direction.

The title salt, **1**, crystallizes in the monoclinic space group *P*2_1_/*c* with one ion pair in the asymmetric unit (Fig. 1 ▸) and consists of C_6_H_11_N_2_^+^ cations and Br^−^ anions. The C8 methyl group is close to coplanar with the imidazole ring [N1—C2—C7—C8 = −8.03 (15)°]. Otherwise, the metrical parameters of the cation agree well with those observed in the other structures involving this species. In the extended structure, the component ions are linked by N—H⋯Br⋯H—N hydrogen bonds (Table 1 ▸) into *C*(8) (Etter *et al.*, 1990 ▸) chains propagating in the *b*-axis direction. The chains are cross-linked in the *c*-axis direction by weak C—H⋯Br hydrogen bonds (Fig. 2 ▸).

## Synthesis and crystallization

The title compound resulted from an attempt to link two 2-ethyl-4-methyl­imidazole rings with a two-carbon chain by the reaction of 2-Et-4-Me-imidazole (6.20 g, 56.3 mmol) with BrCH_2_CH_2_Br (5.32 g, 28.3 mmol) in EtOH at 80°C overnight and several hours at 100°C. Ba(OH)_2_·8H_2_O (8.95 g, 28.3 mmol) was added with ethanol and water and heated to dissolve. On cooling, the mixture was rotovapped down and extracted between water and ether, and the ether layer was evaporated down to 3.1 g of an oil identified as primarily the starting imidazole by NMR. Recovery of about half of the starting imidazole must mean that the oligomer forms preferentially over the dimer. The barium ion was removed from the water layer by titration with H_2_SO_4_ followed by filtration. The solution was rotovapped down to an oil that precipitated a mass of salts on cooling. More crystals of **1** crystallized from the oil over time, and were washed with *i*-PrOH to remove the oil for NMR. NMR of **1** in D_2_O, DSS ref: ^1^H, 1.26 (*t*, 3H), 2.88 (*q*, 2H) (Et), 2.20 (*s*, 3H) (Me), 4.70 (*s*, 1H) (C—H), 6.95 (*s*, 2H) (N—H); ^13^C, 11.8 (Me), 13.3, 21.8 (Et), 117.1 (C—H), 131.4 (4-C), 150.7 (2-C).

## Refinement

Crystal data, data collection and structure refinement details are summarized in Table 2 ▸.

## Supplementary Material

Crystal structure: contains datablock(s) I. DOI: 10.1107/S2414314622011725/hb4418sup1.cif

Structure factors: contains datablock(s) I. DOI: 10.1107/S2414314622011725/hb4418Isup2.hkl

Supporting information file. DOI: 10.1107/S2414314622011725/hb4418Isup3.cml

CCDC reference: 2224817

Additional supporting information:  crystallographic information; 3D view; checkCIF report

## Figures and Tables

**Table 1:** Hydrogen-bond geometry (Å, °)

*D*—H⋯*A*	*D*—H	H⋯*A*	*D*⋯*A*	*D*—H⋯*A*
N1—H1*N*⋯Br^i^	0.829 (17)	2.446 (17)	3.2490 (9)	163.3 (16)
N3—H3*N*⋯Br	0.780 (16)	2.485 (16)	3.2642 (8)	176.6 (16)
C5—H5*A*⋯Br^ii^	0.95	2.93	3.7842 (10)	151
C6—H6*C*⋯Br^iii^	0.98	3.08	3.8349 (11)	135
C7—H7*B*⋯Br^iv^	0.99	2.93	3.8771 (11)	161

Symmetry codes: (i) 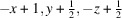 ; (ii) 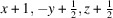 ; (iii) 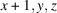 ; (iv) 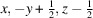 .

**Table 2:** Experimental details

Crystal data	
Chemical formula	C_6_H_11_N_2_^+^·Br^−^
*M*_r_	191.08
Crystal system, space group	Monoclinic, *P*2_1_/*c*
Temperature (K)	100
*a*, *b*, *c* (Å)	6.8432 (6), 15.5962 (13), 7.5748 (7)
β (°)	94.360 (4)
*V* (Å^3^)	806.10 (12)
*Z*	4
Radiation type	Mo *K*α
μ (mm^−1^)	5.02
Crystal size (mm)	0.25 × 0.15 × 0.15

Data collection	
Diffractometer	Bruker APEXII CCD
Absorption correction	Multi-scan (*SADABS*; Krause *et al.*, 2015 ▸)
*T*_min_, *T*_max_	0.571, 0.747
No. of measured, independent and observed [*I* > 2σ(*I*)] reflections	24466, 3936, 3324
*R*_int_	0.027
(sin θ/λ)_max_ (Å^−1^)	0.836

Refinement	
*R*[*F* ^2^ > 2σ(*F* ^2^)], *wR*(*F* ^2^), *S*	0.019, 0.042, 1.03
No. of reflections	3936
No. of parameters	92
H-atom treatment	H atoms treated by a mixture of independent and constrained refinement
Δρ_max_, Δρ_min_ (e Å^−3^)	0.53, −0.36

Computer programs: *APEX2* and *SAINT* (Bruker, 2014 ▸), *SHELXT* (Sheldrick 2015*a* ▸), *SHELXL2018/3* (Sheldrick, 2015*b* ▸) and *SHELXTL* (Sheldrick 2008 ▸).

## full crystallographic data

### Crystal data

**Table:**

C_6_H_11_N_2_^+^·Br^−^	*F*(000) = 384
*M**_r_* = 191.08	*D*_x_ = 1.574 Mg m^−^^3^
Monoclinic, *P*2_1_/*c*	Mo *K*α radiation, λ = 0.71073 Å
*a* = 6.8432 (6) Å	Cell parameters from 9911 reflections
*b* = 15.5962 (13) Å	θ = 3.0–36.4°
*c* = 7.5748 (7) Å	µ = 5.02 mm^−^^1^
β = 94.360 (4)°	*T* = 100 K
*V* = 806.10 (12) Å^3^	Prism, colorless
*Z* = 4	0.25 × 0.15 × 0.15 mm

### Data collection

**Table:**

Bruker APEXII CCD diffractometer	3324 reflections with *I* > 2σ(*I*)
φ and ω scans	*R*_int_ = 0.027
Absorption correction: multi-scan (SADABS; Krause *et al.*, 2015)	θ_max_ = 36.5°, θ_min_ = 2.6°
*T*_min_ = 0.571, *T*_max_ = 0.747	*h* = −11→11
24466 measured reflections	*k* = −26→26
3936 independent reflections	*l* = −12→12

### Refinement

**Table:**

Refinement on *F*^2^	Primary atom site location: structure-invariant direct methods
Least-squares matrix: full	Secondary atom site location: difference Fourier map
*R*[*F*^2^ > 2σ(*F*^2^)] = 0.019	Hydrogen site location: mixed
*wR*(*F*^2^) = 0.042	H atoms treated by a mixture of independent and constrained refinement
*S* = 1.03	*w* = 1/[σ^2^(*F*_o_^2^) + (0.0166*P*)^2^ + 0.2838*P*] where *P* = (*F*_o_^2^ + 2*F*_c_^2^)/3
3936 reflections	(Δ/σ)_max_ < 0.001
92 parameters	Δρ_max_ = 0.53 e Å^−^^3^
0 restraints	Δρ_min_ = −0.36 e Å^−^^3^

### Special details

**Table:**

Geometry. All esds (except the esd in the dihedral angle between two l.s. planes) are estimated using the full covariance matrix. The cell esds are taken into account individually in the estimation of esds in distances, angles and torsion angles; correlations between esds in cell parameters are only used when they are defined by crystal symmetry. An approximate (isotropic) treatment of cell esds is used for estimating esds involving l.s. planes.
Refinement. All hydrogen atoms were located in difference Fourier maps and those attached to N were refined isotropically. Those attached to carbon atoms were refined in idealized geometry using a riding model with with atomic displacement parameters of *U*_iso_(H) = 1.2*U*_eq_(C) [for CH_3_, 1.5*U*_eq_(C)] with C—H distances of 0.95 to 0.99 Å.

### Fractional atomic coordinates and isotropic or equivalent isotropic displacement parameters (Å^2^)

**Table:**

	*x*	*y*	*z*	*U*_iso_*/*U*_eq_	
Br	0.28697 (2)	0.12428 (2)	0.15497 (2)	0.01551 (3)	
N1	0.63750 (12)	0.41897 (5)	0.31428 (11)	0.01531 (14)	
H1N	0.631 (3)	0.4721 (11)	0.314 (2)	0.037 (5)*	
C2	0.49850 (13)	0.36487 (6)	0.25237 (11)	0.01449 (16)	
N3	0.56746 (12)	0.28564 (5)	0.28135 (11)	0.01568 (13)	
H3N	0.503 (2)	0.2457 (9)	0.254 (2)	0.026 (4)*	
C4	0.75365 (14)	0.28880 (6)	0.36671 (13)	0.01609 (15)	
C5	0.79682 (13)	0.37312 (6)	0.38762 (13)	0.01694 (15)	
H5A	0.914514	0.396452	0.442412	0.020*	
C6	0.86990 (17)	0.21085 (7)	0.41511 (15)	0.02302 (19)	
H6A	0.793013	0.172611	0.485620	0.035*	
H6B	0.990828	0.227271	0.484469	0.035*	
H6C	0.902634	0.181162	0.307207	0.035*	
C7	0.30245 (14)	0.38674 (7)	0.16718 (13)	0.01933 (17)	
H7A	0.201752	0.353879	0.225655	0.023*	
H7B	0.296411	0.368801	0.041511	0.023*	
C8	0.25416 (18)	0.48149 (8)	0.17584 (17)	0.0284 (2)	
H8A	0.120458	0.491216	0.123853	0.043*	
H8B	0.346648	0.514173	0.109621	0.043*	
H8C	0.264154	0.500287	0.299648	0.043*	

### Atomic displacement parameters (Å^2^)

**Table:**

	*U*^11^	*U*^22^	*U*^33^	*U*^12^	*U*^13^	*U*^23^
Br	0.01508 (4)	0.01385 (4)	0.01734 (4)	0.00039 (3)	−0.00055 (3)	−0.00153 (3)
N1	0.0149 (3)	0.0141 (3)	0.0167 (3)	−0.0012 (3)	−0.0004 (3)	−0.0013 (3)
C2	0.0152 (4)	0.0147 (4)	0.0135 (3)	−0.0015 (3)	0.0006 (3)	−0.0010 (3)
N3	0.0159 (3)	0.0150 (3)	0.0162 (3)	−0.0026 (3)	0.0014 (3)	−0.0022 (3)
C4	0.0150 (4)	0.0173 (4)	0.0160 (4)	0.0012 (3)	0.0015 (3)	−0.0011 (3)
C5	0.0131 (3)	0.0187 (4)	0.0188 (4)	−0.0006 (3)	−0.0001 (3)	−0.0023 (3)
C6	0.0240 (5)	0.0210 (4)	0.0244 (5)	0.0076 (4)	0.0038 (4)	0.0006 (4)
C7	0.0166 (4)	0.0238 (4)	0.0168 (4)	−0.0007 (3)	−0.0036 (3)	−0.0005 (3)
C8	0.0238 (5)	0.0265 (5)	0.0331 (6)	0.0058 (4)	−0.0089 (4)	0.0014 (4)

### Geometric parameters (Å, º)

**Table:**

N1—C2	1.3299 (12)	C6—H6A	0.9800
N1—C5	1.3844 (13)	C6—H6B	0.9800
N1—H1N	0.829 (17)	C6—H6C	0.9800
C2—N3	1.3351 (12)	C7—C8	1.5167 (16)
C2—C7	1.4836 (13)	C7—H7A	0.9900
N3—C4	1.3851 (12)	C7—H7B	0.9900
N3—H3N	0.780 (16)	C8—H8A	0.9800
C4—C5	1.3545 (14)	C8—H8B	0.9800
C4—C6	1.4836 (14)	C8—H8C	0.9800
C5—H5A	0.9500		
			
C2—N1—C5	109.52 (8)	H6A—C6—H6B	109.5
C2—N1—H1N	126.7 (12)	C4—C6—H6C	109.5
C5—N1—H1N	123.7 (12)	H6A—C6—H6C	109.5
N1—C2—N3	107.15 (8)	H6B—C6—H6C	109.5
N1—C2—C7	127.33 (8)	C2—C7—C8	113.45 (8)
N3—C2—C7	125.52 (8)	C2—C7—H7A	108.9
C2—N3—C4	110.17 (8)	C8—C7—H7A	108.9
C2—N3—H3N	120.7 (12)	C2—C7—H7B	108.9
C4—N3—H3N	129.1 (12)	C8—C7—H7B	108.9
C5—C4—N3	105.90 (8)	H7A—C7—H7B	107.7
C5—C4—C6	131.20 (10)	C7—C8—H8A	109.5
N3—C4—C6	122.89 (9)	C7—C8—H8B	109.5
C4—C5—N1	107.24 (8)	H8A—C8—H8B	109.5
C4—C5—H5A	126.4	C7—C8—H8C	109.5
N1—C5—H5A	126.4	H8A—C8—H8C	109.5
C4—C6—H6A	109.5	H8B—C8—H8C	109.5
C4—C6—H6B	109.5		
			
C5—N1—C2—N3	−1.28 (10)	N3—C4—C5—N1	−0.29 (11)
C5—N1—C2—C7	178.55 (9)	C6—C4—C5—N1	178.35 (10)
N1—C2—N3—C4	1.11 (10)	C2—N1—C5—C4	0.98 (11)
C7—C2—N3—C4	−178.73 (9)	N1—C2—C7—C8	−8.03 (15)
C2—N3—C4—C5	−0.50 (11)	N3—C2—C7—C8	171.77 (10)
C2—N3—C4—C6	−179.27 (9)		

### Hydrogen-bond geometry (Å, º)

**Table:**

*D*—H···*A*	*D*—H	H···*A*	*D*···*A*	*D*—H···*A*
N1—H1*N*···Br^i^	0.829 (17)	2.446 (17)	3.2490 (9)	163.3 (16)
N3—H3*N*···Br	0.780 (16)	2.485 (16)	3.2642 (8)	176.6 (16)
C5—H5*A*···Br^ii^	0.95	2.93	3.7842 (10)	151
C6—H6*C*···Br^iii^	0.98	3.08	3.8349 (11)	135
C7—H7*B*···Br^iv^	0.99	2.93	3.8771 (11)	161

Symmetry codes: (i) −*x*+1, *y*+1/2, −*z*+1/2; (ii) *x*+1, −*y*+1/2, *z*+1/2; (iii) *x*+1, *y*, *z*; (iv) *x*, −*y*+1/2, *z*−1/2.
